# Length of Pace in Horses

**Published:** 1897-02

**Authors:** 


Length of Pace in Horses.—Small horses, just as small men, have often a longer pace than large horses. It depends on the joint-formation and the length of shank in the front-leg, but especially on the degree in which the shoulder-joint can be opened. Slanting and long shoulders with long fore-arms should produce a greater length of pace. According to Polunski and Shindella, the lasting power of the hind-legs contributes to this also. In instantaneous photographs it is easily seen that the front-leg in action is thrown out the further at the instant the leg supporting the weight has reached the highest degree of tension (or stiffness). Therefore, the movement of the fore-leg must set the standard for the length of pace in a given animal. It does not depend upon how high the fore-leg is raised, nor upon how far forward it may be moved, but merely upon how long the other leg can support the weight of the body. Since now the perpendicular distance from the point of the shoulder of the leg upon the ground to the point of the hoof of the same leg exercises great influence, and the length of the legs beneath the shoulder-joint is the most important factor in this, so the length of pace is practically dependent upon the length of the legs. The fore-leg can bear the body longer when the centre of gravity of the horse is further toward the rear. Long-bodied horses have, therefore, an advantage over short ones. The extension of the limbs depends upon the angle which the (pipe?) bone makes with the hip- or huckle-bone. Sloping or slant-joints can be extended wider than straight ones. The shoulder-blade is fixed so movably to the body that a perpendicular as well as a sloping shoulder-blade can describe the necessary arc. We are justified in assuming that the mobility of the shoulders is not exercised to its full capacity while the animal is in motion. An arrangement for measuring the movement of the shoulders was made, but the experimenters were not able to establish a longer pace in the case of horses with greater freedom of mobility in the shoulders. As to whether the length of the legs beneath the shoulder-blade influences the length of pace, it was found that horses with a long pace had a comparatively long fore-arm. No difference in the length of shoulder-blade could be detected between horses with a long pace and those with a short one. Besides, the shoulder-angle —*i.e.*, the angle which a line in the direction of the shoulders forms with the horizontal—is usually 60° to 70°, and is never under 58°. In the hind legs the length of the upper and under arms varies without exerting any influence on the length of pace. Here also the thigh-bone forms an angle of from 68° to 70° with the horizontal line. In conclusion the writer strongly disagrees with the article of Chelchowsky in the *Monatschrift für Thierheilkunde.*

